# Progesterone Treatment Shows Benefit in a Pediatric Model of Moderate to Severe Bilateral Brain Injury

**DOI:** 10.1371/journal.pone.0087252

**Published:** 2014-01-28

**Authors:** Rastafa I. Geddes, Eric A. Sribnick, Iqbal Sayeed, Donald G. Stein

**Affiliations:** Department of Emergency Medicine, Emory University, Atlanta, Georgia, United States of America; University of Pittsburgh, United States of America

## Abstract

**Purpose:**

Controlled cortical impact (CCI) models in adult and aged Sprague-Dawley (SD) rats have been used extensively to study medial prefrontal cortex (mPFC) injury and the effects of post-injury progesterone treatment, but the hormone's effects after traumatic brain injury (TBI) in juvenile animals have not been determined. In the present proof-of-concept study we investigated whether progesterone had neuroprotective effects in a pediatric model of moderate to severe bilateral brain injury.

**Methods:**

Twenty-eight-day old (PND 28) male Sprague Dawley rats received sham (n = 24) or CCI (n = 47) injury and were given progesterone (4, 8, or 16 mg/kg per 100 g body weight) or vehicle injections on post-injury days (PID) 1–7, subjected to behavioral testing from PID 9–27, and analyzed for lesion size at PID 28.

**Results:**

The 8 and 16 mg/kg doses of progesterone were observed to be most beneficial in reducing the effect of CCI on lesion size and behavior in PND 28 male SD rats.

**Conclusion:**

Our findings suggest that a midline CCI injury to the frontal cortex will reliably produce a moderate TBI comparable to what is seen in the adult male rat and that progesterone can ameliorate the injury-induced deficits.

## Introduction

*What is the problem*? Of the estimated 1.7 million traumatic brain injury (TBI) cases in the US each year, almost half a million involve children aged 14 years and younger [1]. Between 2002 and 2006, 92.7% of the children and 59.7% of the adults who visited the emergency department did so due to a TBI-related event [1]. Over the same period, males aged 0 to 4 years had the most emergency department visits, hospitalizations and deaths combined (1,451 per 100,000) [1], making gender and age important predictive factors in pediatric TBI. In fact, from 1997 to 2007, males across all age groups were three times (28.8 per 100,000 population) as likely as females (9.1 per 100,000) to die from a TBI [2].

Since TBI predominantly affects young males, we investigated whether progesterone had neuroprotective effects in a pediatric model of moderate to severe bilateral brain injury in juvenile male Sprague-Dawley (SD) rats. Controlled cortical impact (CCI) models in adult and aged SD rats have been used extensively to study medial prefrontal cortex (mPFC) injury and the effects of post-injury progesterone treatment, but the hormone's effects after traumatic brain injury (TBI) in juvenile animals have not been determined. CCI parameters used in our adult rats resulted in extremely high mortality in our younger subjects. Therefore we modified our injury parameters to create a more moderate impact. We chose to study animals that were 28 days old at the time of injury because in the SD male rat this is the optimal stage of maturation for studying developmental effects of juvenile TBI. The timing of a developmental brain injury can be important in influencing functional outcomes. For instance, the GABAergic system is excitatory in neonatal rats and up to approximately post-natal day 28 (PND 28) [3]–[7], and inhibitory after PND 28, so inflicting an injury followed by a neuroprotective treatment could have a completely different outcome depending on whether the damage is done before, or just after PND 28. Furthermore, by PND 28 the male SD rat has yet to undergo balano-prepubital separation [8], a hallmark of puberty onset in these animals. Finally, the behavioral performance of younger rats (prior to weaning or younger than PND 25) could also be affected by differences in maternal care [9], the social dynamics of dam life [10], [11], and/or an inability to thermoregulate [12], [13]. All these factors can play a role in injury outcome and confound a dose response study/analysis. Measuring all these variables could certainly be the subject of additional experiments in even younger subjects.

*Why propose a pediatric treatment with progesterone*? There is now a substantial literature showing that treatment with progesterone is beneficial in adult rodents after CCI, and the hormone shows promise as a treatment for acute TBI in adult patients [14]–[18]. The multicenter national ProTECT III (NCT00822900) and BHR Pharma's international SyNAPSe® (NCT01143064) Phase III clinical trials are independently evaluating the effects of progesterone treatment on acute TBI in adults, and the Pediatric Emergency Care Research Network (PECARN) is considering a progesterone trial in pediatric patients with moderate to severe TBI [19].

Because progesterone is a potent developmental hormone, before any testing in children is started, we believe it will be critical to determine the safety and efficacy of administering progesterone to the injured developing brain in a rodent model of TBI. A growing literature also demonstrates that age as well as gender may profoundly influence TBI-related functional deficits and histopathology [20]–[24]. Although progesterone has been given at the high end of the dose range (25 mg/kg) to prevent pre-term birth (see for example [25], [26]), the literature suggesting its use as a potential neuroprotective treatment for pediatric TBI is very limited. It has already been shown that exogenous progesterone can treat seizures [27], [28] and alter the pathology of neonatal brain injury [7], [29], [30], but if given too early in development (e.g. in PND 7 and PND 14 animals), it can exacerbate injury. However, it does not have this effect in PND 21 animals [7]. As noted above, the age-related, different outcomes could be due to the fact GABAA is excitatory in early life but later becomes inhibitory. Progesterone is known to upregulate GABA through its metabolite allopregnanolone. If this happens in neonates, its effects could be excitotoxic [3]–[7]. The literature does offer discussions of some potential fetal and early postnatal effects of endogenous and exogenous progesterone or progestins [31], [32], [34], [35], but there is still no clear consensus about progesterone safety in very young brain-injured subjects.

The present study sought to determine whether administration of a 4, 8 or 16 mg/kg dose of progesterone, beginning 1 h post-injury, would be functionally beneficial (or detrimental) in juvenile male rats subjected to bilateral TBI given on PND 28. Goss and colleagues previously demonstrated that moderate (8 and 16 mg/kg) doses, as opposed to a small (4 mg/kg) or a large (32 mg/kg) dose, of progesterone were not detrimental in treating a CCI injury in adult male SD rats [33]. A similar progesterone dose-response study in juvenile rats with injury to the mPFC has not yet been published, so we sought to address this and determine whether progesterone treatment for TBI would be as effective in treating pediatric patients as it is in adults.

## Methods

### Subjects

Seventy-six SD rats (Harlan) were acquired at PND 21 and acclimated to the environment over 2 days. Rats were first weighed on PND 23–25 and daily thereafter. Animals were housed, fed and maintained on a 12-hour reverse light/dark cycle as in Experiment 1. The Institutional Animal Care and Use Committee of Emory University approved the procedures used in this study and the research was conducted in an AAALAC-approved facility (Protocol # 2001801).

### Surgery

Injury was induced on PND 28. Rats were placed in an air-tight induction chamber with oxygen, nitrous oxide, and isoflurane gas (4% induction, 1.5% maintenance, 700 mmHg/min N_2_O, and 500 mmHg/min O_2_). The rats were then mounted in a Kopf stereotaxic device (model 900) equipped with a Mouse and Neonatal Rat Adaptor (model BJK-030). The animal's head was held in place by ear bars and a bite bar and anesthesia was delivered by nose cone. The rats' heads were shaved and sterilized with 70% ethanol and Betadine™ antiseptic solution. Anesthesia levels were monitored closely throughout surgery and were frequently adjusted between 400–700 mmHg/min, based on heart rate and oxygen saturation. In the adult rat CCI-injury model, a 5.0 to 6.0-mm diameter impactor is commonly used [34], but this size proved fatal in the juvenile rats. Here we found that a 4.0-mm diameter impactor induced a survivable, severe contusion injury in the younger rats.

A SurgiVet™ pulse oximeter (model V3304) was placed on the rear paw to monitor and maintain blood SpO_2_ at or above 90%. A heart rate monitor with its sensor attached to the other hind paw was used to observe and maintain a rate ≥300 beats per minute. To prevent hypothermia throughout surgery a homeothermic blanket control unit (Harvard Apparatus, Holliston, MA) was used to monitor and maintain core body temperature (∼37**°**C). Using aseptic techniques, a midline incision was made along the scalp into the skin and fascia covering the skull, exposing the cranium and its bony landmarks including bregma (β) and lambda (λ).

An electric burr drill was used to produce bilateral craniectomies centered 2.0 mm anterior to β and 2.0 mm lateral to the midline (on each side). After craniectomy, injured animals received an injury centered on the midline. Contusions were made to the mPFC using the Impact One™ Stereotaxic CCI instrument (Leica #39463920) designed to produce pneumatic-CCI injury in small animals. The tip of the impactor (4.0 mm diameter) was moved 2.0 mm anterior to β retracted to set the new dorsal–ventral coordinate 3.0 mm ventral to the dura, and then activated to traumatize the cortical tissue. Dwell time was 500 ms and impactor velocity was 2.25 m/s at a pressure of 1.7 psi. Following lesion or sham surgery, the scalp was closed using 4-0 chromic gut sutures. Sham surgeries (n = 24) included all procedures up to and including craniectomy and the steps for closing the incision. After surgery, all rats were maintained on a heating pad, monitored closely and returned to home cages once they regained consciousness [35], [36]. All animals included in the statistical analysis regained consciousness and motor control no more than 5 min after being removed from the stereotaxic device.

### Progesterone solution and injection schedule

Of the 76 rats at the beginning of the experiment, 5 died under surgery. Following surgery, the remaining 71 rats were assigned to an experimental condition based on their baseline data. CCI+ progesterone groups received a dose of 4 (n = 12), 8 (n = 12), or 16 mg/kg (n = 11). Progesterone (4-pregnene-3, 20-dione, Sigma) was dissolved in 22.5% 2-hydroxyropyl-β-cyclodextrin (HBC) (Sigma). Progesterone doses smaller (4 mg/kg) and larger (16 mg/kg) than 8 mg/kg were included in this dose-response study because previous work found that 8 mg/kg of progesterone was the most effective in adult and rats [33] and humans [16]. Each dose was administered at an equal volume (0.2 ml/100 gm b.wt.) relative to body weight. Vehicle-treated rats with CCI (n = 12) and vehicle-treated rats with sham surgeries (n = 12) received equal volumes of HBC relative to body weight. A total of 9 injections were administered to each animal in all experimental conditions at the following post-injury times: 1, 3, and 24 h, and 2, 3, 4, 5, 6, and 7 days. With the exception of the first dose, which was administered intraperitoneally to ensure more rapid absorption following injury, all injections were given subcutaneously [33]. It is important to note that the 8^th^ and 9^th^ injections of progesterone were tapered (one half and one one-quarter of the original dose, respectively) to avoid withdrawal effects [37], [38]. Progesterone was prepared just prior to surgery and then again on the 4^th^ post-injury night.

### Behavioral testing

Rats were tested before surgery on rotarod, grip strength and open field performance to obtain baseline data. All post-surgery testing was delayed for 1 week, during which rats received daily vehicle or progesterone injections as described above. Animal IDs were coded to keep experimenters blind to group identity throughout behavioral testing and histological analysis.

#### Rotarod testing

Balance and motor coordination were assessed under red light using an accelerating Exconomex™ rotarod (Columbus Instruments, Columbus, OH). Before injury, rats were given initial habituation training and baseline testing. For habituation training, rats were placed onto a stationary rod for at least 3 min, then slowly habituated to the rotating rod from a starting speed of 1 rpm, which was accelerated to 30 rpm over 5 min. A baseline score was obtained on PND 27 and rats were again tested on PID 9, 15, 21, 25 and 27. Rats were scored on latency to fall off the rod (maximum score was 300 s), and each testing day consisted of three trials separated by a 5-min break. Scores from the three trials for each day were averaged.

#### Locomotor activity (open field) testing

Testing for spontaneous hyperactivity was done under red light in a quiet environment on PND 27 for baseline, and on PID 9, 15, 21, 25 and 27. For each trial, the OptoVarimex activity monitoring system (Columbus Instruments) was used to test 2 animals (cage mates) simultaneously in individual boxes. Rats were placed in the left lower corner of the activity box with the recording apparatus on. Exactly 5 min later the computer stopped recording movements, ensuring that all tests were the same length regardless of start time. Animals were returned to their home cages at the end of testing. The activity boxes were cleaned with 70% ethanol and dried between trials.

#### Spatial navigation performance and memory in the MWM

MWM tests were conducted under dim white light in a white plastic pool measuring 135 cm in diameter, in a room with numerous visual cues outside the pool, including a blue curtain, lamps, and 1 gray and 1 white 2×2 ft sheet of construction paper on adjacent (north and east) walls in the room. The pool was filled with water at ∼21°C and made opaque by adding white nontoxic water-soluble tempera paint (Crayola). A platform of white 12×12 cm Plexiglas was submerged 1.0 cm below the surface of the water in the southwest quadrant of the tank. An overhead camera and computer-assisted tracking system (San Diego Instruments Inc., San Diego, CA) recorded the rat's position in the maze, swim distance, heading, and time taken to find the platform. Because tracking was based on contrast, each rat's head and neck area was smeared with nontoxic black marker before the animal was released into the pool.

Beginning on PID 10, all rats were tested for acquisition in the MWM. Each rat received two trials per day, separated by a 5-min interval. There are four arrows on the outside of the pool indicating four equally spaced positions (N, S, E and W). During Run 1 and Run 2, the animals were placed in the pool at West and South starting points, respectively. On PID 10–15 the MWM platform was located in the NE quadrant, which consists of the cone-shaped space between the North and the East starting positions. On PID 18–20 the platform was located in the SE quadrant. A trial consisted of placing the subject in the pool at the W position, facing the wall at the beginning of the trial. The rats were allowed to swim until they reached the platform or until 90 sec had elapsed. When rats were unable to locate the MWM platform within the allotted time, the experimenter led the rat to the platform. Rats were allowed to remain on the platform for 20 sec and then removed from the pool. After 5 min, subjects were again released into the tank, but this time from the S position, and allowed to swim to the platform. Between and after trials, animals were placed in holding cages in front of an air heater before being returned to their home cages.

#### Anxiety-like behavior in the Elevated Plus Maze (EPM)

The wooden plus-shaped maze is 50 cm above the ground, with each 10×90 cm arm joined at a 90-degree angle. One pair of opposing arms is surrounded on either side by 40-cm-high walls, while the other pair is open. The EPM is used to evaluate anxiety (via amount of time spent in the open arms) and locomotor activity (via total number of arm entries), as well as rearing behaviors [39], [40]. An arm entry was counted only when the rat crossed the center square line into a given arm resulting in both front and hind limbs being in the arm at the same time. When a rat fell off the maze, it was returned to the start position in the center square and the fall recorded. Individual averages of all trial data were obtained for each rat. The maze was cleaned with 70% ethanol and allowed to dry between trials.

Testing was conducted under red light in a quiet environment. Baseline EPM data was obtained between PND 25 and PND 27 and rats were tested twice post-surgery (on PID 9 and 17). Each trial lasted 5 min, and the total number of open arm entries (or crosses over the center square with both front and hind paws) was then reported as percent of visits to the open vs. closed arms. The total arm entries and percent visits to open and closed arms were then analyzed for lesion and drug effects.

### Histology

At 4 weeks post-injury, all animals were deeply anesthetized and their brains perfused with saline, then with 10% formalin, and then extracted for histological analysis. After post-fixation in 10% formalin for 24–48 h, brains were immersed successively for 2–3 days each in 15%, 20%, and 30% sucrose solution in dH**_2_**O, then removed and stored at −80**°**C until sectioned. The forebrain was cut into 20-µm sections on a cryostat and stored at −80°C on 1% gelatin coated (subbed) slides. Slides were stained in 0.1% cresyl violet solution (0.1 g cresyl violet acetate and 0.3 ml of glacial acetic acid dissolved in 100 ml dH_2_O) for 10 min at 45°C and then rinsed in distilled water. Slides were then washed in 95% alcohol for 5–10 min, in 100% alcohol (2×5 min), and then in xylene (3×5 min). Sections were mounted with xylene-based cytoseal. Lesion surface area was identified and analyzed from Nissl-stained sections between 4.5 and −0.5 mm from β (see section 2.4.2. for details) using the Image J™ System (Media Cybernetics, Silver Spring, MD), a free computer-assisted image analysis program.

Following immersion in 30% sucrose, brains were stored at −80°C. Each rat brain was then removed from the −80°C freezer and placed in the cryostat at −20°C for 20 minutes before being sliced into 20-micron thick sections. Six evenly spaced anterior-to-posterior sections (4.5, 3.5, 2.5, 1.5, 0.5 mm anterior to β, and −0.5 mm posterior to β were then stained with hematoxylin and eosin (H&E), examined for evidence of CCI-induced tissue damage across groups [41], and selected for lesion cavity measurements. Stained slides were scanned using Silverfast Pathscan software (PathScan Enabler IV, Meyer Instruments, Houston TX). ImageJ™ was used to analyze the scanned images. The percentage of injured tissue from a single section was calculated by tracing the perimeter of the injury and determining its surface area, dividing this by an estimate of the total surface area of the section (taken by tracing both the remaining tissue and the estimated perimeter of the necrotic cavity), and multiplying by 100. The estimated percentage of damaged tissue from H&E-stained sections for each rat was determined by averaging the percentage of damaged tissue across six sections, which spanned 4 mm (i.e., +4.5, +3.5, +2.5, +1.5, −0.5 mm to β). In the sham group, rats were excluded because of slight but noticeable drill-induced injury during the craniectomy. We wanted to reduce any potential variance due to this factor.

#### Qualitative observations on animals surviving the TBI

**Figure 1:**
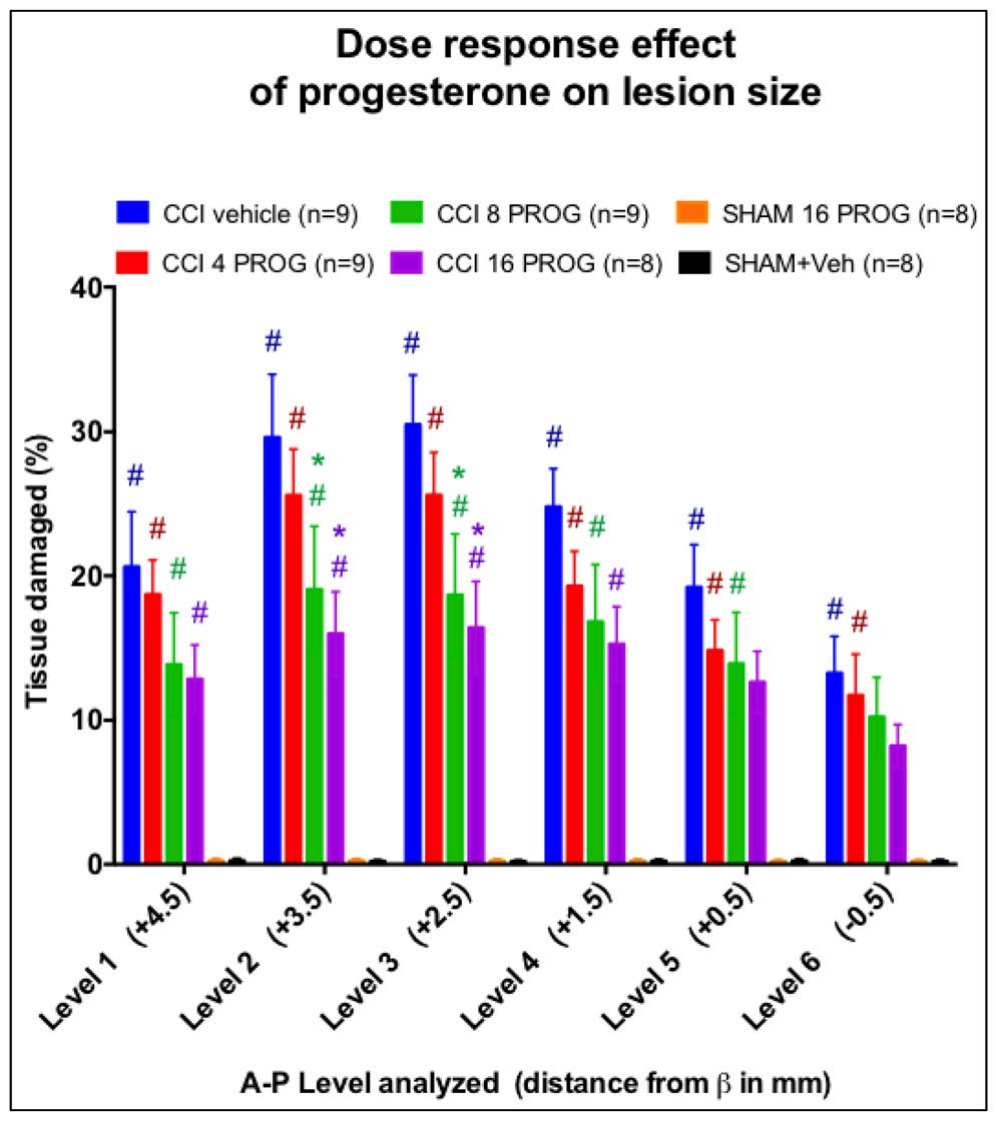
Lesion reconstruction analysis of CCI rat pups treated with progesterone vs. vehicle. Mean percent (±SEM) of volumetric tissue loss at the six anterior to posterior (A/P) levels examined between groups at 4 weeks post-injury. * =  significant difference between the CCI vehicle-treated group and CCI rats given (8 or 16 mg/kg dose of) progesterone. # =  significant difference between CCI group and both sham groups regardless of progesterone dose administered (*p*<0.05). Values are mean ±SEM (n = 8–9/group). PROG =  progesterone.

**Figure 2:**
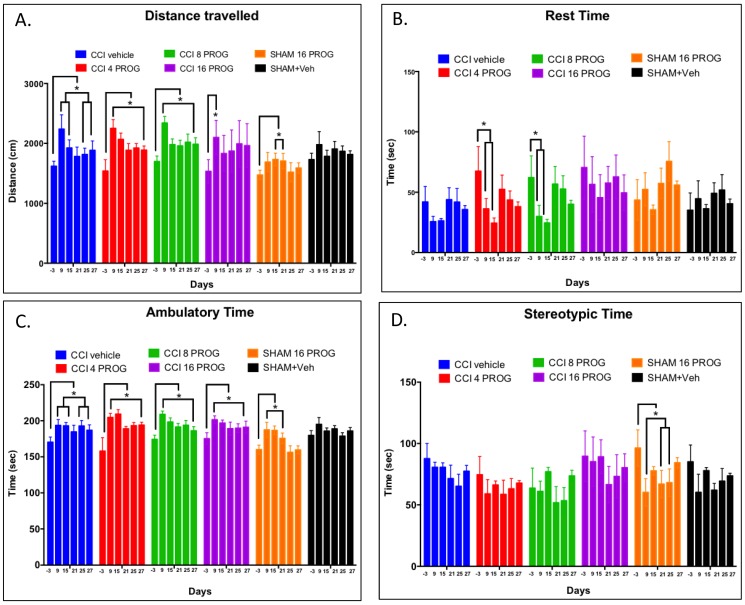
Dose-response effect on Spontaneous Activity test. Mean distance travelled (**a**), mean time spent at rest (**b**), Ambulatory responses (**c**) and stereotypic responses (**d**). Although CCI had a significant effect on spontaneous activity across test days, across the group there was no observed significant effect of Lesion/Treatment. * =  significant difference from baseline within each group. Values are mean ±SEM (n = 8–9). PROG =  progesterone.

**Figure 3:**
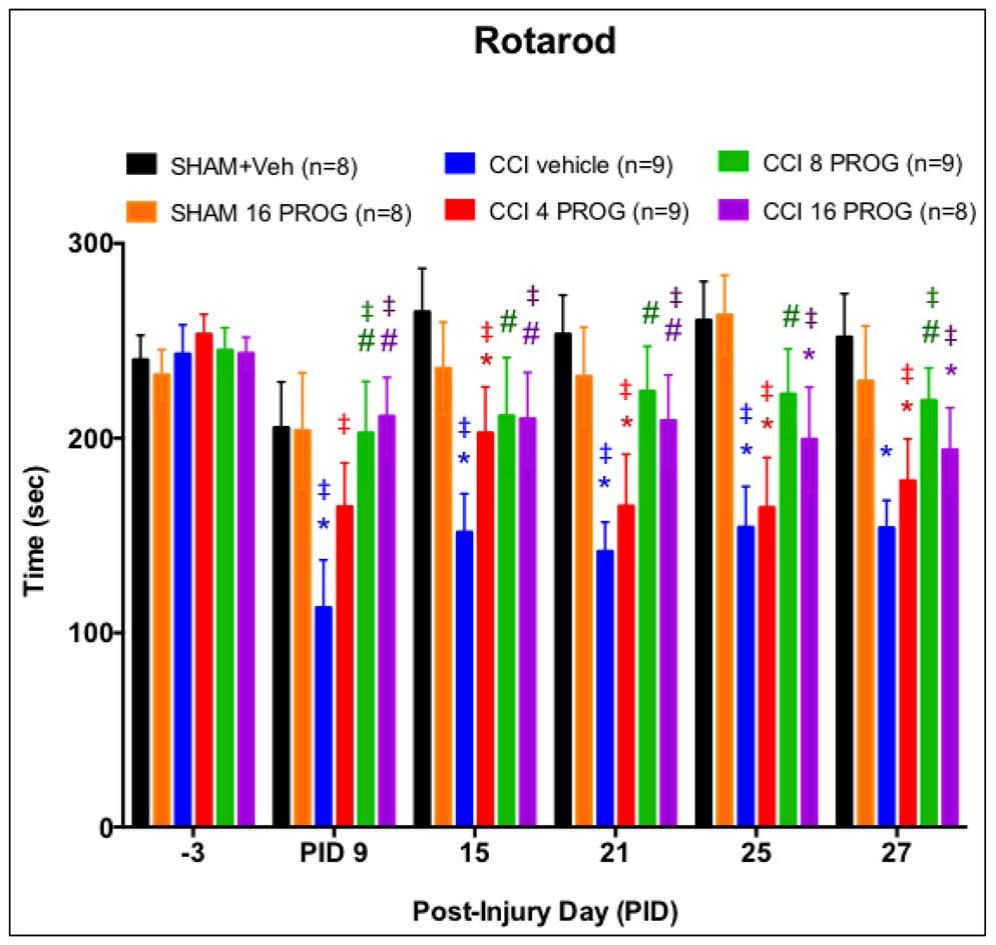
Dose-response effect of progesterone treatment on vestibulomotor function. Progesterone-treated rats showed improvement in balancing and walking on the rotarod tasks. * =  different from sham + vehicle; # =  significantly different from CCI+ vehicle (*p*'s<0.05). ‡ =  significant difference from baseline within each group (color coded). Values are mean ±SEM (n = 8–9). PROG =  progesterone.

**Figure 4:**
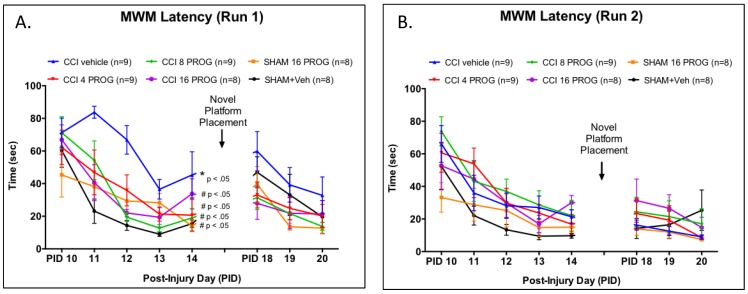
Dose-response effect of progesterone on learning and memory task as assessed by latency to locate the MWM platform. During Run 1 (**a**) on the acquisition phase there was a significant effect of CCI injury and all three doses of progesterone proved beneficial by decreasing the mean latency latency to find a hidden platform in the MWM compared to the CCI+ vehicle treated group. There was no clear effect of CCI injury on Run 2 (**b**) or during the reversal phase (p>0.05). * =  mean values are significantly different from sham; n = 8–9. PROG =  progesterone.

**Figure 5:**
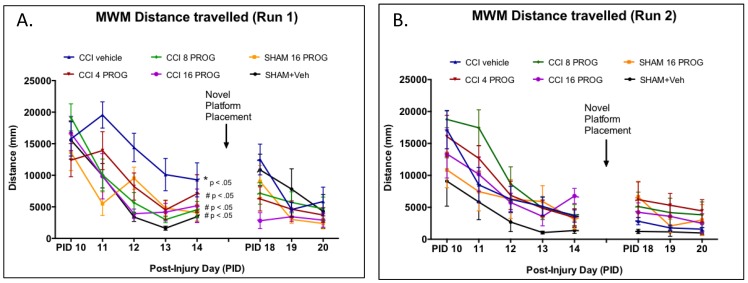
Dose-response effect of progesterone on learning and memory tasks as assessed by distance travelled in the MWM. During Run 1 (**a**) in the acquisition phase there was a significant effect of CCI injury and all three doses of progesterone proved beneficial by decreasing the mean distance travelled by rats seeking the hidden platform in the MWM, compared to the CCI+ vehicle-treated group. While the sham + vehicle-treated group appears to do better, given the performance and variability of the other groups there was no clear difference between vehicle-treated sham and CCI rats on Run 2 (**b**) or during the reversal phase (p>0.05). * =  mean significantly different from sham + vehicle; # =  significantly different from CCI+ vehicle. n = 8–9. PROG =  progesterone.

**Figure 6:**
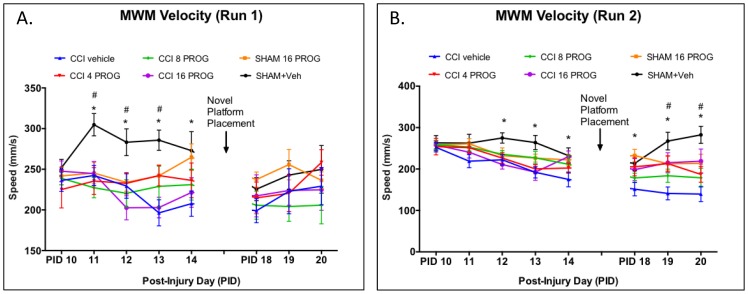
Dose-response effect of progesterone on mean velocity in the MWM. During Run 1 (**a**) in the acquisition phase there was a significant effect of CCI injury. While progesterone treatment eventually improved performance, when collapsed across trials the 4–16 mg/kg CCI+PROG groups were not statistically different from CCI+ vehicle-treated rats. During Run 2 (**b**) swim speed for the CCI+ vehicle group compared to Sham Vehicle was significantly different. PROG (16 mg/kg)+CCI groups showed the most improvement on swim speed during reverse phase learning. * =  significantly different from CCI+ vehicle; # =  significantly different from CCI+8 mg/kg (*p*'s<.05). n = 8–9. PROG =  progesterone.

After surgery the CCI+16 progesterone group had 11 rats and the remaining 5 groups had 12 rats each. Of the 24 sham rats, the data for 8 were removed from final analysis. The reasons for removal included: failure to meet the criterion of 180 ms on the rotarod during baseline testing (n = 2); evidence of unreliable data during post-surgery testing (n = 2) due to attempts to escape the apparatus or hugging the rod (thus missing the sensor); and moderate unintentional injury to brain from the drill bit during craniectomy (n = 4). The data for 16 out of 24 sham-operated rats (n = 8 in the sham plus vehicle and sham plus progesterone groups) are presented in Figures 1–6. Of the 47 remaining rats with CCI injury, 3 failed to meet rotarod baseline criteria and another 3 repeatedly attempted to escape the apparatus rather than run on the wheel. Finally, 6 rats died after surgery but before all behavioral tests were completed. Thus, the data for 35 out of 47 rats with CCI injury are also presented in Figures 1–6, with n = 9 rats each in the CCI plus vehicle, the CCI plus 4-mg/kg and the CCI plus 8-mg/kg progesterone. The CCI plus 16-mg/kg progesterone group had n = 8 rats.

### Statistical Analysis

A mixed factorial ANOVA for repeated measures was performed on the behavioral results and lesion reconstruction data, which were expressed as the mean ±SEM. Mean comparisons were used for *post-hoc* analyses of repeated ANOVAs. Independent paired t-tests were also used to compare the differences between baseline (pre-injury) and post-injury data when data was normally distributed. Statistical significance was established at *p*≤0.05. Based on a delta-value of 1.5 (using latency to find the hidden platform during Run 1 on the MWM task) we calculated the sample sizes and power needed to reject the null hypothesis (of no differences among the treatment groups relative to untreated CCI-injured controls) to achieve >80% power to detect a 30% difference (medium effect). The number of rats per group at these criteria was determined to be at least 9.

## Results

### Lesion (tissue) reconstruction analysis

Briefly, the six sections from each rat brain were photographed with an Epson scanner (and ImagePro™ software). Group means of percent of damaged tissue averaged across six anterior-posterior sections were used to determine overall lesion size. Sham rats with a visible drill-induced lesion (n = 4), were excluded from behavioral statistical analysis. Tissue sections from the remaining sham rats had no observable damage. Results from the reconstruction analysis of sham and CCI-injured lesions treated with vehicle or progesterone are shown in Figure 1. There was a significant main effect of Lesion/Treatment on estimated lesion size (F(5,45) = 30.31, *p*<0.05). *Post-hoc* comparisons further revealed significant differences between sham and CCI (*p*'s<0.05). Finally, while significant differences were observed between progesterone and vehicle-treated CCI rats (*p*<0.05), no differences were detected between 4-, 8- and 16-mg/kg doses of progesterone (*p*>0.05).

### Physiological and functional effects of PND 28 CCI-induced injury

#### Body Weight

One-way ANOVA with repeated measures on PND or PID was conducted on the mean pre- and post-injury body weights. There was no main effect of Lesion/Treatment on mean body weight (F(5,45) = .811, *p*>0.05) (Figure S1). As might be expected, there was, however, a main effect of PID on mean body weight (F(28,1260) = 5282.0, *p*<.001). *Post-hoc* pair-wise comparisons indicated that the group mean body weights were significantly increased over time (*p*<0.01), except for the first two days post-surgery (*p*>0.05).

#### Spontaneous locomotor activity

The open field test was performed prior to CCI or sham surgeries and then again on the 9^th^, 15^th^, 21^st^, 25^th^ and 27^th^ PID. Dependent measures were ambulatory distance travelled (cm), ambulatory/active time (sec), rest time (sec), and time spent eliciting stereotypic behaviors (sec). ANOVA revealed a main effect of (and interaction between) Lesion/Treatment and Test Day (F's>1, *p*<0.05). The analysis also revealed that at baseline there were no significant differences in ambulatory distance, ambulatory time or rest time between the 6 groups tested (*p's*>0.05). Baseline behavioral data on these parameters were collected prior to surgery and (along with body weight) used to assign rats to the 6 groups so that baseline measures did not differ among groups. After surgery and treatment, Lesion/Treatment had a significant effect on ambulatory time (F(1,45) = 5.34, *p*<0.05), but not rest time or stereotypic behaviors (F's<1, *p*'s>0.05); the effect on ambulatory distance was also found to be significant (F(1,45) = 3.92, *p* = 0.05). Finally, while no progesterone dose effect was observed in the 2×4×6 ANOVA (F(3,45) = 1.99, *p*>0.05), the interaction between Lesion and progesterone dose had a significant effect on ambulatory time (F(1,45) = 4.20, *p*<0.046), but not on ambulatory distance (F(1,45) = 3.05, *p*>0.088) (Fig. 2).

As seen in Figure 2a, independent *post-hoc* comparisons of the group means found that regardless of progesterone dose (0, 4, 8, 16 mg/kg), CCI produced a significant rise in ambulatory distance on PID 9 (2239 cm, 2251 cm, 2341 cm, 2333 cm, respectively), compared to the sham-plus-vehicle (1979 cm) or the sham-plus-progesterone (1689 cm) treatment group (*p*'s<0.05). On PID 15, however, CCI animals returned to baseline activity levels (∼1637 cm, *p*'s>0.05). By PID 25 and on PID 27, the 16-mg/kg dose of progesterone increased ambulatory distance to 2220 cm and 2187 cm, respectively. In the intact rats progesterone treatment produced activity levels similar to baseline at 1520 and 1589 cm, respectively. CCI had a transient, and the 16-mg/kg dose of progesterone had a delayed, effect on hyperactivity in the open field.

#### Elevated Plus Maze

Repeated measures ANOVA were performed on EPM behaviors. The independent measures were Lesion and Drug dose and the dependent measures were the percent of entries in the open vs. closed arms and the total number of crossings. The ANOVA revealed no significant main effect of, or interactions between, Lesion and Drug dose, on the number of arm entries, percent of entries in each arm or total crossings (F's<1, *p*>0.05; data not shown).

#### Rotarod

Activity on the rotarod was measured as the latency to fall off the rotating/accelerating rod over a 300-sec period. A repeated 6×6 (Lesion/Treatment x Test Days) measures ANOVA, with repeated measure on PID, revealed a main effect of Lesion/Treatment on rotarod performance (F(5,45) = 3.87, *p*<0.05). *Post-hoc* analysis further revealed that rotarod performance during baseline testing was not different among the groups (*p*>0.05). As indicated in Figure 3, CCI injury and progesterone treatment had a significant effect on rotarod performance. CCI rats treated with vehicle and the group given 4 mg/kg progesterone were significantly worse than sham plus vehicle on PID 9, 21, 25 and 27 (* = *p*<0.05). As shown in Figure 3, the 8 and 16 mg/kg doses of progesterone reduced CCI injury-induced deficits. These two progesterone groups had significantly better rotorod scores than the CCI vehicle (*) and 4 mg/kg progesterone group (#) and were not different from the sham groups.

#### Spatial navigation and cognition in the MWM

Mean latency to reach the MWM platform within 90 sec during acquisition and novel platform placement learning, distance travelled, and velocity served as the dependent measures. Group latency scores from Runs 1 and 2 are shown in Figure 4a–b and were analyzed using a 6×2×9 (Lesion/Treatment x Run x Test Day) mixed factorial ANOVA, with repeated measures on PID.

While all groups were relatively slow in finding the platform during their first trial on PID 10, there was a significant main effect of Lesion/Treatment, Run and Test day (F's>1, *p*'s<0.05). On Run 1 during acquisition training, when collapsed across PID, CCI injury significantly increased the time taken to reach the MWM platform in rats treated with vehicle, whereas all doses of progesterone diminished the effects of CCI injury on MWM performance. These three groups were not significantly different from sham controls in this measure. Compared to sham rats, CCI vehicle-treated rats (Fig. 4a) took significantly longer to find the MWM platform (*p*<0.05; Fig. 5a), travelled longer distances (*p*<0.05), and (Fig. 6a) swam at a slower speed (*p*<0.05) during acquisition.

As shown in Figure 4b, sham vehicle-treated rats were the most efficient in using the Run 1 experience to find the MWM platform in Run 2 (which was conducted 5 min after Run 1). In Run 2, despite having no difference in the distance travelled during Run 2 (Figure 5b), sham vehicle-treated rats maintained a steadier and higher swim velocity than the other groups (Figure 6b). Placing the MWM platform in a novel quadrant was transiently disruptive to the performance pattern observed in sham rats at the end of acquisition (PID 14). Overall, CCI had no effect on acquisition learning and performance on the three novel platform placement trials. Taken together our data show that the CCI injury was sufficient to produce observable histopathological, behavioral and cognitive deficits in male PND 28 rat pups. Finally, poor performance on one task was NOT predictive of poor performance on other tasks. The correlation (r = −.246) between vestibulomotor performance and dependent spatial learning was not significant (*p*>0.05), suggesting low predictive value across these two distinct behavioral tasks.

## Discussion

Although the neuroprotective effects of progesterone have been successfully demonstrated following TBI in adult and aged male and female subjects, we show here that it may also be beneficial in 3–4 week-old male rat pups. We consider this work an important step in evaluating whether progesterone is a good candidate for clinical testing in pediatric brain injury. It is important to note that negative side effects at any dose of progesterone used here were minimal in the rodents with a brain injury. The dosing parameters we used are also consistent with those used in numerous studies with adult subjects. Since we observed no disruptive effects of the treatments in the present study within the dosing regimen and under the conditions of these experiments, we think that the treatment is likely to be safe when used in juvenile/adolescent animals. However, it should be noted that the question of whether progesterone should be used as a neuroprotective treatment in even younger neonatal subjects is still not resolved. We know that one recent study showed that both progesterone and its metabolite, allopregnanolone, exacerbated the outcome of hypoxic injury when given to PND 7 and PND 14 rats, but not in PND 21 animals [7]. These detrimental effects are likely due to the fact that increasing the levels of GABA_A_ in very young subjects actually causes excitotoxicity in both males and females. However, in slightly older animals, while PROG and ALLO still increase GABA_A_, there is a developmental transition of the neurotransmitter from excitatory to inhibitory. Given these findings it is clear that further toxicity/safety/efficacy studies should be done if pediatric treatments are to be considered for very young children with hypoxic or traumatic brain injuries.

In this study, 28-day old male rat pups were given a short course of progesterone or vehicle treatment after having received either craniectomy or a CCI directly to the mPFC. Behavioral testing on rotarod, spontaneous activity, EPM and MWM began 2 days after the last progesterone injection and lasted up to up to 1 month post-injury. We found that: (1) The 8 and 16 mg/kg doses of progesterone were most beneficial and consistent with studies from our laboratory [33] and others [42]. (2) Progesterone treatment resulted in dose-dependent decreases in the extent of damage. Compared to CCI lesion plus vehicle, when averaged over the six coronal sections analyzed for injury, the mean lesion volumes of the progesterone-treated rats with a CCI injury were smaller.

As for behavior, (3) the brain injury led to only slightly decreased visits to the open arm in the EPM at 9 days post-injury. (4) In the Open Field, the 16-mg/kg dose of progesterone increased the distance travelled in CCI rats, suggesting that these animals were less anxious. (5) Vehicle-treated CCI resulted in significant deficits in the rats' ability to remain on the rotorod, and in spatial-cue learning performance in the MWM. (6) Interestingly for the rotarod and MWM tests, the 16-mg/kg dose of progesterone was found to be beneficial for some rats with CCI injury and had no effect on others. Taken together, our findings suggest that short-term administration of the optimal dose of progesterone may be a viable therapeutic treatment for pediatric TBI in males. Additional studies should be done to determine whether similar outcomes might be obtained in pre-pubescent female laboratory rats.

### CCI injury and progesterone treatment: Effects on pathophysiology

Immediately after surgery, we documented the extent of hemorrhage, hematoma, and herniation as a result of craniectomies and/or CCI. We found that the group differences in these parameters did not seem to predict injury severity or progesterone effectiveness on recovery of functions regardless of the doses used. We did not see any sustained (>3 days) effects of injury or treatment on body weight in our animals. With respect to metabolic activity such as weight gain and activity, the young rats in our experiment appeared to be much more resilient to CCI than can be expected in more mature animals with the same type and extent of damage.

### CCI injury and progesterone treatment: Effects on lesion extent and severity

The midline CCI injury to the mPFC in one-month-old rats produced a cavity highly consistent with a moderate contusion seen in adult rodents [35], [43]. In the Hoffman, et al. study [35], CCI injury to the mPFC in the adult male SD rat produced lesion cavities that, at 18 days post-injury, were largest (≥4 mm^2^) at the most anterior (+4.7, +3.7 and +2.7 mm to β) sections we examined for neuronal lesion volume.

We found that progesterone at 4, 8, and 16 mg/kg reduced infarct cavity size (by ∼25%), despite variable functional effects. Our vehicle-treated CCI PND 28 rats had >20% damage, while the progesterone-treated CCI groups had <15% damage. Roof and colleagues [44] were the first to demonstrate that in adult male and female SD rats, seven injections of 4 mg/kg progesterone could significantly reduce edema and increase neuronal cell counts in the lateral and medial dorsal thalamic nuclei [44]. Goss and colleagues [33] later demonstrated that post-injury treatment with 8 and 16 mg/kg, as opposed to 32 mg/kg, doses of progesterone in adult male SD rats were optimal for functional recovery after injury to the mPFC [33]. In Roof's study, despite improvement in MWM performance, rats given 4 mg/kg progesterone or vehicle had injuries that represented 46.07% and 40.89% of the total coronal section, respectively [44]. In Goss's study, despite the ability of progesterone to reduce bilateral somatosensory neglect and improve MWM and EPM performance, at PID 25, 20% of the tissue section was damaged regardless of treatment (vehicle or progesterone). Progesterone did not reduce lesion size in these experiments.

It is tempting to conjecture that progesterone's effect on lesion cavity size is age-dependent, but it should be noted that almost half (4 out of 9) of the vehicle-treated CCI rats had relatively smaller cystic infarcts (2–3 mm in diameter). Importantly, the behavioral deficits in these animals did not differ from those of the other rats in the same group. Future studies will need to determine whether the reduction in lesion cavity size can be explained by age or gender/hormonal dependency on neuronal sparing or aberrant sprouting in response to pediatric brain injury. Notably, in non-injured rats, the 16-mg/kg dose of progesterone suppressed the distance travelled in the Open Field test and decreased swim speed in MWM learning. This is not surprising, since relatively high doses of progesterone can be used as an anesthetic and anxiolytic in human and animal subjects 29 [45], [46].

## General Conclusion

Here we report that in an animal model of contusion injury to the brain, short-term treatment with progesterone in moderate to high doses given in the acute stage of the injury to one-month-old, pre-pubescent, male rats did not produce any serious detrimental outcomes and was effective in enhancing functional recovery on a number of behavioral parameters involving sensory, motor and cognitive performance. We believe it is essential to conduct a similar dose-response study in female rat pups.

One question that could be raised is whether giving progesterone prior to an injury is relevant to defining the window for neuroprotection following a TBI. We do not believe this to be the case. Both in the laboratory and in clinical practice, progesterone “pretreatment” may have distinct effects from progesterone “post-treatment.” At least two published reports have demonstrated that high levels of progesterone (corresponding to ∼50 ng/ml) given before sustaining a CCI or a middle cerebral artery occlusion (MCAO) were ineffective in ovariectomized adult female rodents [47], [48]. In contrast, the dose, duration, and timing of progesterone *post-treatment* needed for optimal beneficial effects in adult female and male rats with CCI injury has been demonstrated in a number of papers by our laboratory [33], [36], [49], [50].

More recently, Uysal and colleagues [30] demonstrated that a combination therapeutic treatment of 8 mg/kg progesterone and 150 mg/kg magnesium sulfate (MgSO4) administered immediately after a unilateral weight drop-induced TBI on PND 7 significantly (1) reduced apoptosis in the hippocampus and the prefrontal cortex, and (2) decreased deficits in MWM learning and memory. Here we report similar effects. In our hands, an 8 mg/kg dose of progesterone increased the rate of learning in the MWM (when tested at 3 weeks post-injury). We also found that 8 mg/kg of progesterone was as beneficial as the 4 and 16 mg/kg doses in reducing infarct size at 1 month recovery. In another recent study, a single dose of progesterone in immature (PND 7) rats was found to be effective in reducing anxiety and increasing neuronal density in the prefrontal cortex, hippocampus and amygdala [29]. Together, these data suggest that in at least two distinct models of pediatric TBI, progesterone has beneficial effects on trauma-induced histopathology and cognitive dysfunction.

Of the 86 clinical trials for pediatric brain injury, only 5 have tested a potentially neuroprotective agent at the acute stage of injury. Currently, there are no clinically approved agents that can be given to children with TBI, so testing for safety and efficacy trial in young, brain-injured laboratory animals is an important step.

## Supporting Information

Figure S1 **Dose-response effect of progesterone on weight.** Mean body weight (gm) showing weight changes in each group. No significant differences in body weight between groups (*p*>0.05) were observed. Values are mean ±SEM (n = 8–9/group). PROG =  progesterone.
(TIFF)
