# Supplementary figures and images for: Progesterone Treatment Shows Benefit in a Pediatric Model of Moderate to Severe Bilateral Brain Injury
Source: PLoS One. 2014 Jan 28;9(1):e87252. doi: 10.1371/journal.pone.0087252 (PMC3904994; doi:10.1371/journal.pone.0087252)

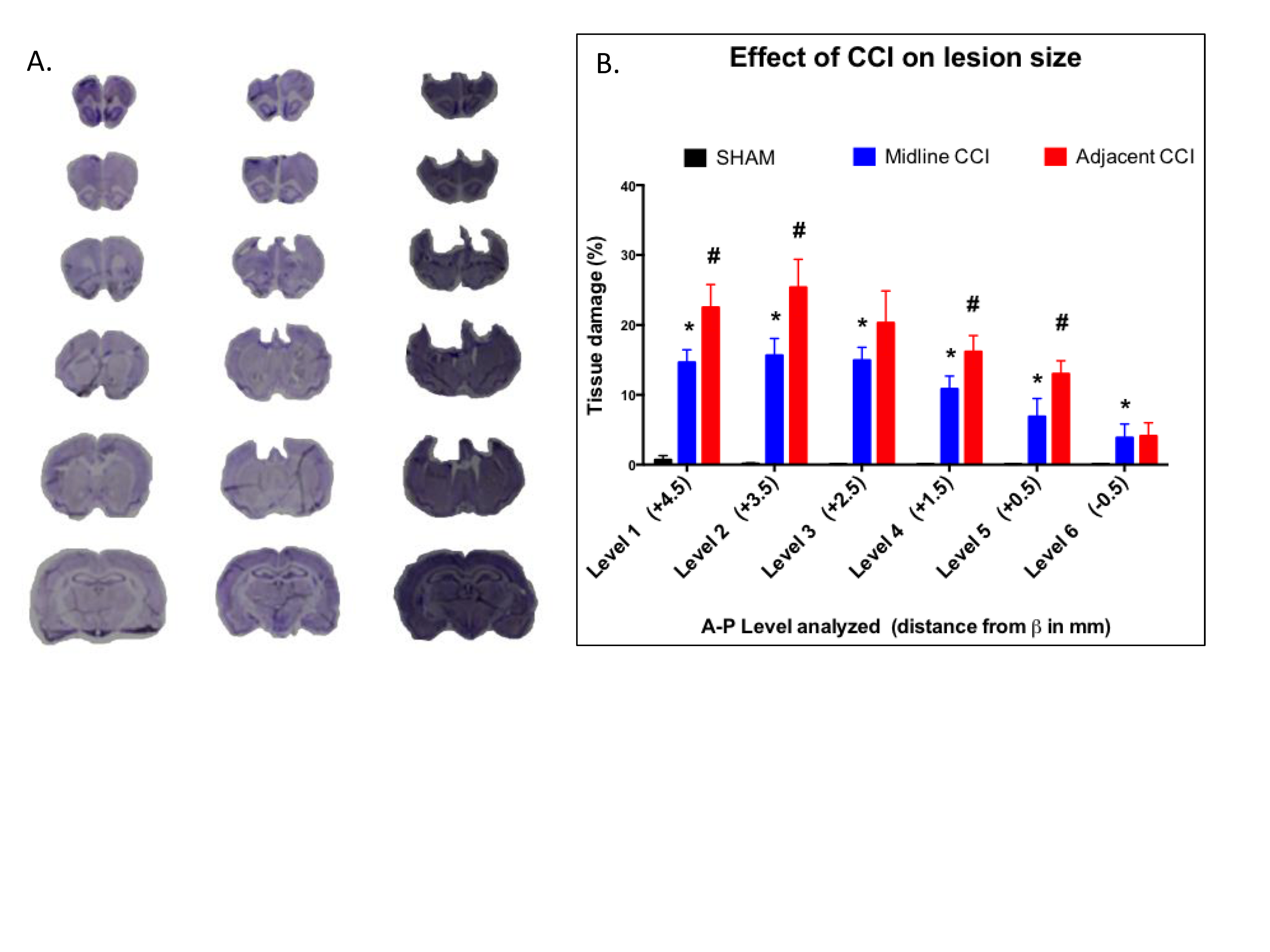

Supplement: Figure S1 — Dose-response effect of progesterone on weight. Mean body weight (gm) showing weight changes in each group. No significant differences in body weight between groups (p>0.05) were observed. Values are mean ±SEM (n = 8–9/group). PROG = progesterone. (TIFF) [file pone.0087252.s001.tif]
